# The inverse relation between risks and benefits: The impact of individual differences in information processing style

**DOI:** 10.1371/journal.pone.0255569

**Published:** 2021-08-09

**Authors:** Patrycja Sleboda, Carl Johan Lagerkvist

**Affiliations:** Department of Economics, Swedish University of Agricultural Sciences, Uppsala, Sweden; Newcastle University, School of Natural and Environmental Sciences, UNITED KINGDOM

## Abstract

Existing research shows that evaluations of the risks and benefits of various hazards (i.e., technologies and activities) are inversely related. The affect heuristic explains the negative relation between risks and benefits, as based on the strength of positive or negative affect associated with a hazard. Research on the affect heuristic previously investigated under which conditions people judge risk and benefits independently, focusing on expertise as a factor that might exempt from inversely related judgements of risk and benefits. Measurements within Dual Process Theories have been found to be associated with rational, analytical decision making and accurate judgments. In this paper we investigated the extent to which rational information processing styles can predict the risk-benefit relation of technologies in a medical and food applications and whether the attitudes influence the strength or direction of the relationship. Using the Need for Cognition Scale (NFC), a psychometric-based risk scale and an explicit measure of attitude, in a representative sample of 3228 Swedes, we found that the high NFC group judged the risks and benefits of technologies to be inversely related. In contrast, the low NFC group judged the risks and benefits to be positively related. These results were confirmed across all studied technologies by applying moderation analysis. We discuss the results in light of recent research on cognitive processing and polarization over technologies’ risks.

## 1. Introduction

Existing research has demonstrated that evaluations of the risks and benefits of various hazards (i.e., technologies or activities) are not independent of each other. Studies have typically documented an inverse relationship between risks and benefits, in which hazards are either judged as high in danger and low in benefits, or low in danger and high in benefits [1–6]. This inverse risk-benefit relation has been examined in relation to numerous hazards, including environmental hazards [1, 7] and new technologies [8], as well as in judgements in the area of finance [9–11]. For example, Fischhoff and collaborators [1] found that motorcycles and nuclear power are judged as very low in benefits and very high in risks, while antibiotics and vaccinations are perceived as high in benefits and low in risks.

The inverse relation in risk and benefit judgements was determined to be formed on the basis of feelings towards hazards. Alhakami and Slovic [2] found a negative relation between the risks and the benefits of several hazards (e.g., pesticides) to be linked to the strength of positive or negative affect associated with the hazard. Finucane and co-authors [3] verified this relationship, proposing the term *affect heuristic* to explain the underlying mechanism of the inverse relationship between risks and benefits. Moreover, a longitudinal study with a two measure 2-year apart has found that risk and benefit judgments are moderately stable and likely to be based on the affect heuristic [12].

The concept of the affect heuristic resides in the theoretical framework of dual information processing [13], which comes in various forms and with various labels i.e., dual-process, dual systems, but always two systems are emphasized. These distinctions were formally locked up into theories according to which the human mind is composed of two information processing systems usually called System 1 and System 2, as labeled by Stanovich and West [14]. Labels of these systems have varied. For example, Epstein [15] labeled those two systems Experiential—Rational, Sloman [16] and Smith and DeCoster [17] called System 1 Associative and System 2 Rule-based, while Tversky and Kahneman [18] Intuitive and Extensional. They do not exactly correspond in all cases, but in general System 1, is related to intuitive, affective, heuristics processing [19]. System 1 “has” the ability to make rapid interaction and read intentions. System 2 is analytical, and reason based; it is also called analytic intelligence, a rational system which is considered to be under control. Authors agree that people use System 1 more often than System 2. System 1 is assumed to constitute a root of cognitive biases, inducing over(under)estimation of risk, and therefore leads to inaccurate evaluations, as compared to evaluations that require the analytical, deliberative form of processing (System 2).

The inverse relation in risk and benefit perception is assumed to originate in a rapid, heuristic, System 1 processing [20]. Kahneman and Frederick [21] pointed out that the affect heuristic is a basic mechanism that guides heuristic judgements and leads to attribute substitutions.

Prior research has investigated under which conditions people make less biased judgements in risk and benefits evaluations. For example, research on the affect heuristic has investigated whether individual differences in expertise can exempt the inversely related judgements of risk and benefit. Specifically, researchers have expected that experts, when judging risks and benefits of technologies, will be more likely to relay on their knowledge and experience and therefore make less biased but more rational judgements. Some studies have reported experts and laypeople to differ in their risk perception, for example when judging chemical products, nuclear power, police work, surgery, electric power, X-rays and swimming [22, 23], mountain climbing [23], hunting, bicycles, and spray cans [22], as well as in the inefficacy of health services and the storage of medical equipment [24], other researchers documented no differences between experts and the public in risk perception [7, 8, 25]. Thus, evidence of the impact of expertise on risk-benefit judgements is inconclusive [26, 27]. Another promising factor in debiasing perception of the risk-benefit relation might be individual differences in information processing as the extend research has reported that individuals with dominant rational information processing system are less prompt to biased judgements [28, 29]. The role of rational information processing in judgements of risks and benefits was recently examined by Skagerlund and collaborators [19] who have expected that high scores on the cognitive reflection test [29] to be less linked to the affect heuristic while more related to rational evaluation of risks and benefits. In study 2 authors have correlation between CRT scores and risk-benefit index [19]. However, at the time of data collection there was no research that directly examined the impact of individual differences in information processing in relation to risk-benefit judgements of hazards. Thus, in the next paragraph we show how we addressed those limitations.

Various measures, rooted in cognitive or social psychology, were designed to distinguish dominant information processing. One such frequently used self-reported measure is the Need For Cognition (NFC) scale [30]. Individuals with a high NFC are performing better when solving cognitive reflection test [29], were found to be less likely to make bias-based judgements [28]. Specifically, individuals with high NFC scores were less influenced by anchors [31] or stereotypical judgements. Moreover, NFC has been found to be an accurate predictor of various academic achievement outcomes, such as solving arithmetic problems and anagrams, as well as performance on trivia tests and college coursework [32]. Some information processing styles measures incorporated NFC in their structures. For example, Epstein proposed the Cognitive-Experiential Self Theory [15], which incorporated the NFC scale as a “rationality” subscale of his Rational-Experiential Inventory (REI) [33, 34]. This has been found to be an effective predictor of rational decision-making. Liberali and collaborators found that NFC was positively correlated with a numeracy scale [35] and the Raven Advanced Progressive Matrices test [36]. NFC has also been shown to be an accurate predictor of performance consistent with normative rules in tasks that are often used to measure rational behavior, such as the missing-a-flight vignette, the thematic and abstract versions of the Wason task, and the jelly bean task [37]. On more general level NFC was found to be a good predictor of the transitivity of preferences [38]. Given the above, we aim to answer (RQ1) whether individual differences in rational information processing relate to risk and benefit judgements of technologies. We expect, (H1) individuals with higher scores on NFC to be less prompt to inversely related judgments of risk and benefits. We also expect, (H2) low scores on NFC to be directly related to inversely related judgements of technologies’ risks and benefits. Moreover, we aimed to determine (RQ2) whether the inverse relation is conditional upon information processing style and affect and, if so, if this effect varies across different technologies. We aim to test whether (RQ3) attitudes (which in this study were used as proxy for affect) influence the strength or direction of relation between information processing style and risk-benefit judgements.

### 1.1. Public perception of modern technologies

The acceptance of modern technologies is central for technological development. Specifically, the extant research with focus on modern technologies within food applications, such as genetically modified crops, has reported that perceptions of risks and benefits, general attitudes, and knowledge constitute major determinants of consumers’ acceptance of modern technologies in food applications [39–42]. The review by Frewer and collaborators [43] found that technologies categorized as ‘bioactive’ typically relate to concerns about the unpredictability of effects. Furthermore, food that is perceived as ‘unnatural’ (e.g., due to pesticides or additives) was found to be unlikely to prompt a high level of public rejection [43]. A more recent investigation, however, suggested that artificial food additives do engender public concern [44]. Notably, for biotechnology, evidence concerning consumer response and acceptance remains unclear. A meta-study did not find salient differences about geographical area as an impact on consumer aversion towards biotechnology for food production [45]. Moreover, previous research indicates that public acceptance exists, in particular for GM food, when providing direct benefits to consumers [46, 47]. Some reports persist, however, that identify a negative attitude towards gene technology for plant breeding across different countries (see studies [48, 49]).

In contrast, the majority of the public in Europe, Canada, and the U.S. support the medical application of gene technology for stem-cells [50]. However, other kinds of medical applications of modern technologies, such as vaccinations, prompt public concern and a hesitant attitude [51, 52]. Additional research is requisite on risk and benefit perceptions of modern technology, as this constitutes a critical factor in technology acceptance.

In the present study, we examined the explanatory impact of individual differences in information processing style on the (inverse)-relation between risks and benefits across five modern technologies in the area of food and medical applications: (1) gene technology for plant breeding; (2) pesticides; (3) food additives–“E”-numbers; (4) gene technology for stem cells; and (5) vaccinations for humans.

The selected items comprise types of technologies with specific food and medical applications. All of them, however, represent a similar level of generality. In particular, we refer to vaccinations for humans, in general, but not to specific vaccinations, and similarly for food additives–“E”-numbers and pesticides. Consequently, we selected gene technology for plant breeding, in which gene editing and gene modification are specific, separate methods. Similarly, we addressed gene technology for stem cells, but did not refer to its specific methods (e.g., for embryonic stem cells, adult stem cells, or induced stem cells).

## 2. Method

### 2.1. Sample

This study was carried out as an Internet survey after initial pre-testing, i.e., a survey was administered on a small sample of approximately 10 subjects before a full-scale study in order to identify any problems, such as an administrating time that was too long or unclear wording. A sample of 3,228 Swedish residents (1,622 female) with a mean age of 41.6 (SD = 13.7, ranging from 15 to 74) completed the entire survey (out of 3,243 that were recruited by a marketing research company). Participants were panelists of the Nepa marketing search company. Panelists of this company received an invitation to participate in a study with a 7 days quarantine default period, during which time they will not receive any new invitations. The average incentive for the panelist was 0,1 Euro per minute, which in the case of this study was approximately 2 Euro (approximately 21 SEK for participation). The incentives were calculated based on the average time of the survey, which was measured in the pre-testing survey involving 10 participants. Participants in the actual study, therefore, know in advance about the money that they could receive for participation. The research complies with the Swedish Ethical Review Authority and the need for ethical consent was waived. There were no misleading questions in the survey. Respondents were assured that they could withdraw at any point of the study, without the need to provide any explanation and without any consequences. No question was design to cause any discomfort to respondents. The demographic characteristics of the final sample were compared to the official Swedish Statistics agency [53]. The sample consisted of 50.2% women, while 49.8% of the Swedish population, in general, is female. The sample was also comprised of more respondents from 25 to 54 years than the Swedish population, in general (66.9% vs. 47.7%). The group from 15 to 24 was slightly underrepresented (11.50% vs. 13.70%), with a slight overrepresentation of the 55 to 64 age group (17.6% vs. 14.02%). The gender proportion in the age groups was similar in the 55 to 64 age group (51% of females in our sample compared to 50% in the Swedish population as a whole), as well as in the 25 to 54 age group (49.1% vs. 49.2%). Women were overrepresented in the younger age group of 15 to 24 (59.9% vs. 48.4%). Additionally, we asked participants about their education: 51.3% reported higher education (as compared to 42% in the Swedish population), 40.6% high school education, and 7.1% primary school. 41.1% of our participants were from an urban area of 150,000 or more people, 37.4% were from other urban areas, and 21.5% were from the countryside. 48% of our participants reported a monthly gross salary of 40,000 SEK or less (as compared to 50% in the Swedish population). We took no further action to weigh these differences in our statistical analysis, as they were very small.

### 2.2. Materials and procedure

On the first page of the survey, participants were informed that they were part of a data collection process conducted by a university. They were further informed that the aim of the study was to understand perceptions of various activities and technologies. They were also told that the study will take approximately 20 min, and that it is completely voluntary and not associated with any risk. All of the instructions were given in the Swedish language. After the first page participants moved to risk questionnaires and were asked to rate each technology (each on a separate page) on 16-item risk scale. One technology at the time was presented in random order (e.g., pesticides) and without any additional information about any of the presented technologies participants were asked to rate it on the following scale. After completing rating about first technology, they moved to the next page which presented the next technology and the 16-item questionnaire for it. That was repeated until participants rated all 5 technologies. Next, attitude questions about each of the technology were presented and at last participants filled Need for Cognition scale.

#### 2.2.1. Risk questionnaire

The measure developed by Savadori and colleagues [8] was adapted to measure risk perceptions of five technologies, three related to food i.e., gene technology for plant breeding (henceforth, GT); pesticides; food additives–“E”-numbers and two medical technology applications, i.e. stem cells, and vaccinations for humans. Participants were presented with the Swedish translation of the questionnaire. Participants evaluated the risks for each technology with a 16-item questionnaire (see S1 File), among which harm and benefit questions were asked separately in relation to humans and the environment, e.g., *How much harm will derive from this application to humans (to the environment)*? *To what extent will humans (the environment) benefit from this application*? Participants were presented with the Swedish translation of the questionnaire and were asked to rate each technology on a scale from 1 to 11 [8]. Technologies were presented in random order.

#### 2.2.2. General attitudes

Participants were asked to state their general attitude towards each technology (e.g., *Please state on a scale from 0 to 100 what is your general attitude towards pesticides*) using a 0–100 scale (0 = very negative; 100 = very positive). For attitude questions, technologies were presented in random order.

Both classical theories of attitude and modern theories address attitude expression in relation to affect. For example, Sehimmack and Crites [54] refer to the valence of attitude as an expression of affective component. Eagly and Chai ken [55] describe attitude as “a psychological tendency that is expressed by evaluating a particular entity with some degree of favor or disfavor” (p. 1). Attitude was also referred to in a similar manner by Peters and Slovic [56], as attitudes towards technologies or activities “appear to be oriented by means of both affect and cognition” (pp. 1448–1449). Therefore, on this basis, general attitudes are treated as a proxy for affect, understood here as an overall degree of positivity or negativity toward the attitude’s object, in this case each technology (e.g., Ajzen [57]).

#### 2.2.3. Need for Cognition

To control for individual differences in information processing style, we utilized one of the most commonly used self-report scales—the Need for Cognition (NFC) developed by Cacioppo and Petty in 1982.

**Fig 1:**
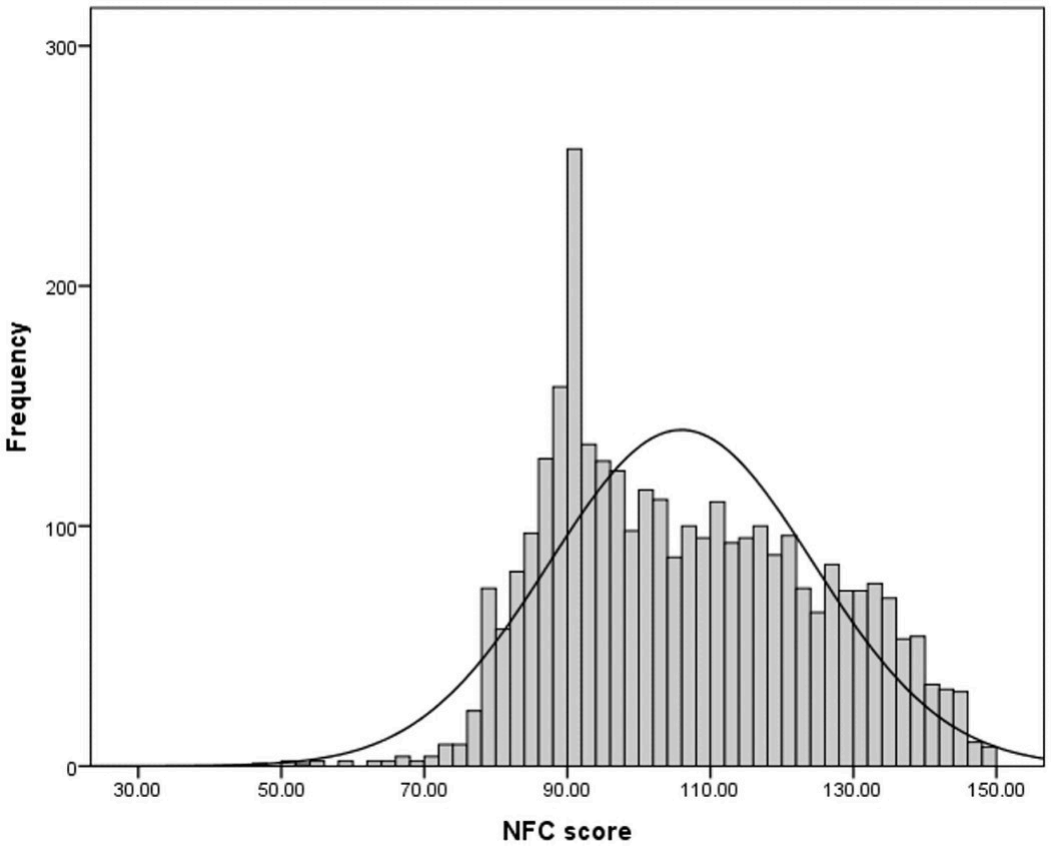
Distribution of NFC scores.

The Swedish adaptation and validation of the original Need for Cognition scale [30], published by Dornic and collaborators [58], was used (for a recent validation, see Jonsson, Stenlund, & Johnsson [59]). The scale consists of 30 items on a 5-point, Likert-type scale (1 = strongly disagree, 3 = neutral, and 5 = strongly agree). Twelve of the statements were designed to indicate positive attitudes toward engaging and enjoyable thinking, while 18 indicated negative attitudes. Items that indicated negative attitudes required reverse scoring in order to conclude that high scores indicated a high NFC. The mean score of the 30-item scale was 105.93 (SD = 18.39), with scores ranging from 47 to 140 (skewness = 0.287, kurtosis = -0.78). This corroborates results from previous studies [58, 60]. Cronbach’s alpha coefficient was α = 0.91. The distribution of the scores on NFC is presented in Fig 1. The correlation matrix with NFC and demographics can be find in the S1 Table in S1 File. For the detailed relation between study variables, that is NFC, attitudes and measures of inverse relation with the demographics (gender, income, and education) please see S4-S6 Tables in S1 File.

## 3. Results

### 3.1. The relationship between harms and benefits by categorization due to need for cognition

To answer first research question whether individual differences in high vs. low NFC relates to perception of risk and benefits participants were assigned to two groups based on their NFC score: low (N = 500) or high (N = 635) in NFC. The division criterion, following previous research by Petty et al. [61], was the sample mean and SD (one SD below or above the mean [62]). Tables 1 and 2 show the mean and correlation coefficients between the dimensions related to harms and benefits to humans and the environment for each technology, separately for the two NFC groups. For means of benefits and harms for the entire sample, for each of the hazards (both for humans and the environment), and their correlation and general attitudes, please see S2 Table in S1 File.

**Table 1:** Mean benefit and harm judgements for food and medical application of technologies by low Need for Cognition (NFC) (N = 500; upper) and high Need for Cognition (NFC) (N = 635; lower).

	**Low NFC**				
	Benefit to humans	Harm to humans	Benefit to environment	Harm to environment	General attitude
**GT for plant breeding**	7.04 (2.46)	7.04 (2.31)	6.73 (2.53)	7.10 (2.39)	51.30 (28.09)
**GT for stem cells**	7.36 (2.28)	6.89 (2.39)	6.73 (2.48)	6.72 (2.39)	55.40 (27.36)
**Pesticides**	6.97 (2.39)	7.39 (2.26)	6.55 (2.75)	7.45 (2.31)	49.82 (29.39)
**Food additives—“E”-numbers**	6.69 (2.58)	7.18 (2.28)	6.44 (2.72)	7.08 (2.30)	48.26 (28.64)
**Vaccination**	7.37 (2.41)	6.79 (2.49)	6.84 (2.53)	6.69 (2.52)	59.26 (27.28)
	**High NFC**				
	Benefit to humans	Harm to humans	Benefit to environment	Harm to environment	General attitude
**GT for plant breeding**	7.18 (2.80)	5.82 (2.83)	5.38 (2.92)	6.20 (2.87)	44.96 (32.14)
**GT for stem cells**	8.21(2.47)	4.72 (2.48)	5.30 (2.71)	4.47 (2.55)	65.50 (29.79)
**Pesticides**	5.97 (2.66)	7.62 (2.52)	3.46 (2.49)	8.18 (2.52)	29.12 (26.94)
**Food additives—“E”-numbers**	5.31 (2.84)	6.38 (2.78)	3.72 (2.50)	5.99 (2.76)	32.49 (28.16)
**Vaccination**	8.69 (2.54)	4.35 (2.70)	5.51 (2.96)	4.18 (2.60)	75.67 (29.04)

Note: *GT* denotes gene technology. Harms and benefits were evaluated on a scale from 1 to 11; attitudes were estimated on a scale from 0 (very negative) to 100 (very positive); the number in brackets after the mean values represents SD.

**Table 2:** Pearson’s correlations between benefit and harm for the low and high NFC groups; the significance of differences in pairs of correlation coefficients tested using fisher r to z transformation.

	Benefit/harm to humans			Benefit/harm to environment		
	Low NFC	High NFC	Difference (z-value)	Low NFC	High NFC	Difference (z-value)
**GT for plant breeding**	**.357** **	**-.506** **	15.53***	**.406** **	**-.584** **	18.34***
**GT for stem cells**	**.443** **	**-.502** **	17.15***	**.626** **	**-.154** **	14.85***
**Pesticides**	**.421** **	**-.386** **	14.28***	**.363** **	**-.502** **	15.55***
**Food additives—“E”-numbers**	**.422** **	**-.492** **	16.49***	**.504** **	**-.330** **	14.97***
**Vaccination**	**.338** **	**-.596** **	17.33***	**.601** **	**-.181** **	14.64***

* = p < 0.05,

** = p < 0.01,

*** = p < 0.001

Table 1 shows that individuals characterized as high in NFC perceived GT for plant breeding, stem cells, and vaccination as high in benefit and low in harm. The same pattern was observed for the human and environmental harm-benefit relation, respectively. In contrast, the high NFC group perceived pesticides and food additives–“E”-numbers to be high in harm and low in benefit. Furthermore, the correlations of harm-benefit perceptions of all technologies within the high NFC group were negative (Table 2).

In contrast, the group which was low in NFC perceived the benefits and harms of all technologies, both with humans and environmental consequences just above the middle of each scale. In addition, correlations between harms and benefits across all technologies for both humans and environmental consequences were positive.

**Fig 2:**
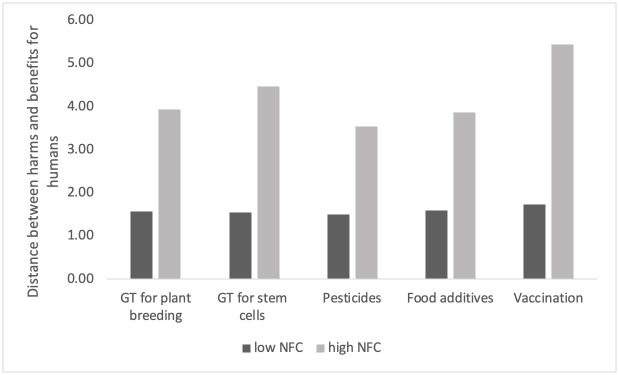
Distance between harms and benefits for humans for two groups: Low and high in NFC.

**Fig 3:**
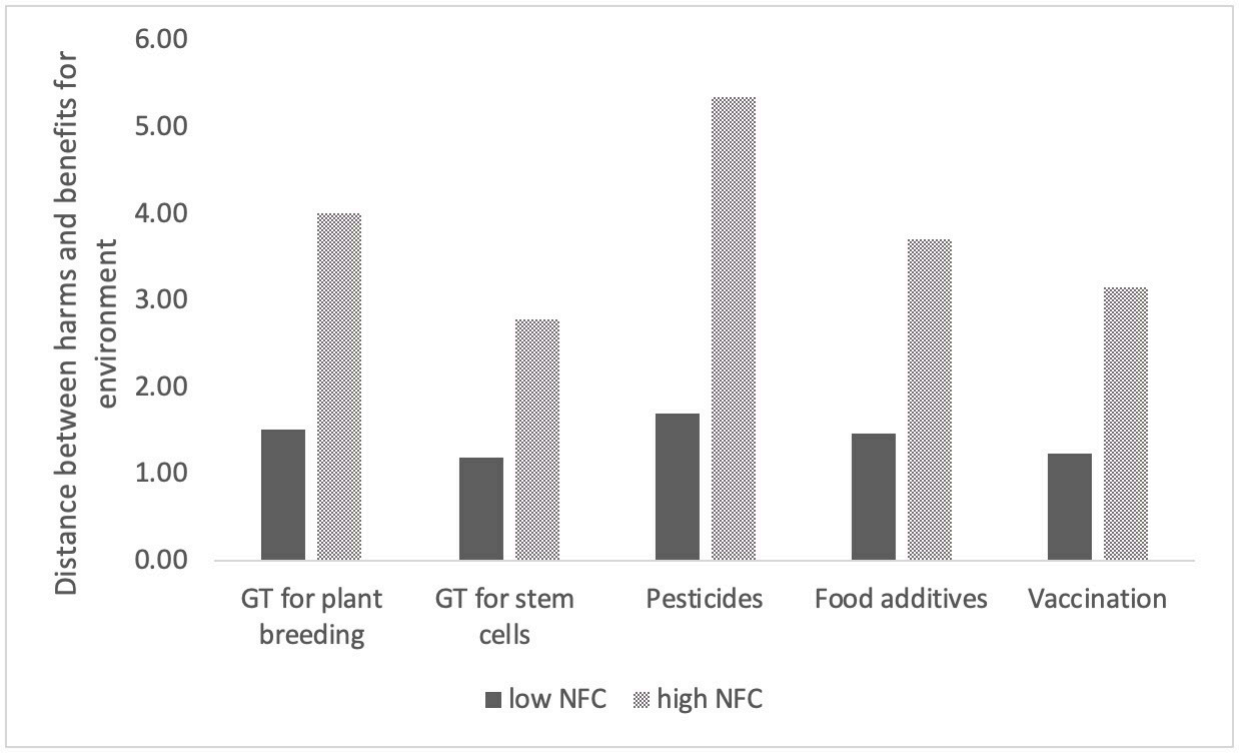
Distance between harms and benefits for the environment for two groups: Low and high in NFC.

Since correlations express only the general degree to which harms and benefits, on average, are related within each group, we followed Alhakami and Slovic [2], and in the next step calculated the distances between harms and benefits. For each participant, we calculated the absolute differences between perceived harms and benefits for each technology (i.e., for humans and the environment, respectively). Note, harms and benefits were evaluated on a scale from 1 to 11. Thus, the higher the distance, the greater the difference in the harm and benefit relation, i.e., either harm was evaluated as low and benefit as high, or harm as high and benefit as low. The distance measures were consistent with the correlations. As can be seen in Figs 2 and 3, participants with high NFC were found to perceive greater distances between harms and benefits for both types of technologies (i.e., for humans (Fig 2) and for the environment (Fig 3)). Regarding distances between harms and benefits for humans, the greatest difference between high and low NFC groups was found for vaccination (D_highNFC_ = 5.44, SD = 3.36; D_lowNFC_ = 1.74, SD = 2.35) and GT for stem cells (D_highNFC_ = 4.46, SD = 3.26; D_lowNFC_ = 1.54, SD = 2.05); whereas, for GT for plant breeding (D_highNFC_ = 3.93, SD = 3.23; D_lowNFC_ = 1.58, SD = 2.21), pesticides (D_highNFC_ = 3.53, SD = 2.98; D_lowNFC_ = 1.51, SD = 2.01) and food additives–“E”-numbers (D_highNFC_ = 3.87, SD = 3.11; D_lowNFC_ = 1.58, SD = 2.06), the differences in distance were similar.

The differences in perception of harms and benefits for the environment were the most salient for pesticides (D_highNFC_ = 5.36, SD = 3.52; D_lowNFC_ = 1.71, SD = 2.46). In addition, the differences in distances between low and high NFC groups were significant for both harm-benefit distances for humans (F (5, 1129) = 125.79, p < .001; Wilk’s Λ = 0.642, partial η2 = 0.36) and the environment (F (5, 1129) = 102.87, p < .001; Wilk’s Λ = 0.687, partial η2 = 0.31), as confirmed with multivariate ANOVA.

### 3.2. The moderating role of attitude and technology

**Fig 4:**
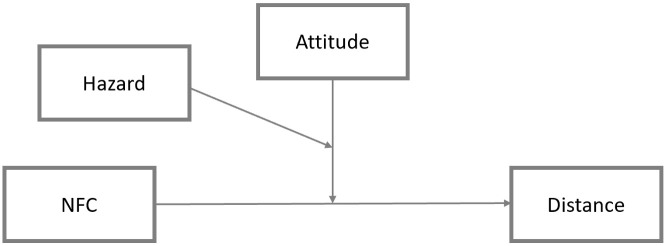
Conceptual model: Three-way interaction effect of attitude (W) and technology (Z) on the relationship between NFC (X) and distance between harms and benefits (Y).

In the next step (RQ2 and RQ3), to examine whether attitudes influence the strength or direction in the relation between individual differences and risk-benefit evaluation, we employed the three-way interaction model regression based moderated moderation analysis [63]. There are no serious multicollinearity concerns in any of the presented model. We have followed the typical correlations thresholds of 0.8 and which was not met in any of the correlation of the included variables. For more details, please read Disatnik and Sivan [64] to learn more on what has been called the illusion of multicollinearity in moderations. This analysis was employed on the entire sample of participants (N = 3,228) with NFC as continuous variable. Fig 4 shows the conceptual research model used to test whether the relationship between NFC (continuous, independent variable) and risk-benefit distance (dependent variable) is moderated by attitude (in this study used as a proxy for affect), and whether this moderation, in turn, is dependent on technology type as a secondary (categorical) moderator [65]. For technologies’ means of attitudes for the entire sample and with respect to low and high NFC groups, please see S3 Table in S1 File.

**Fig 5:**
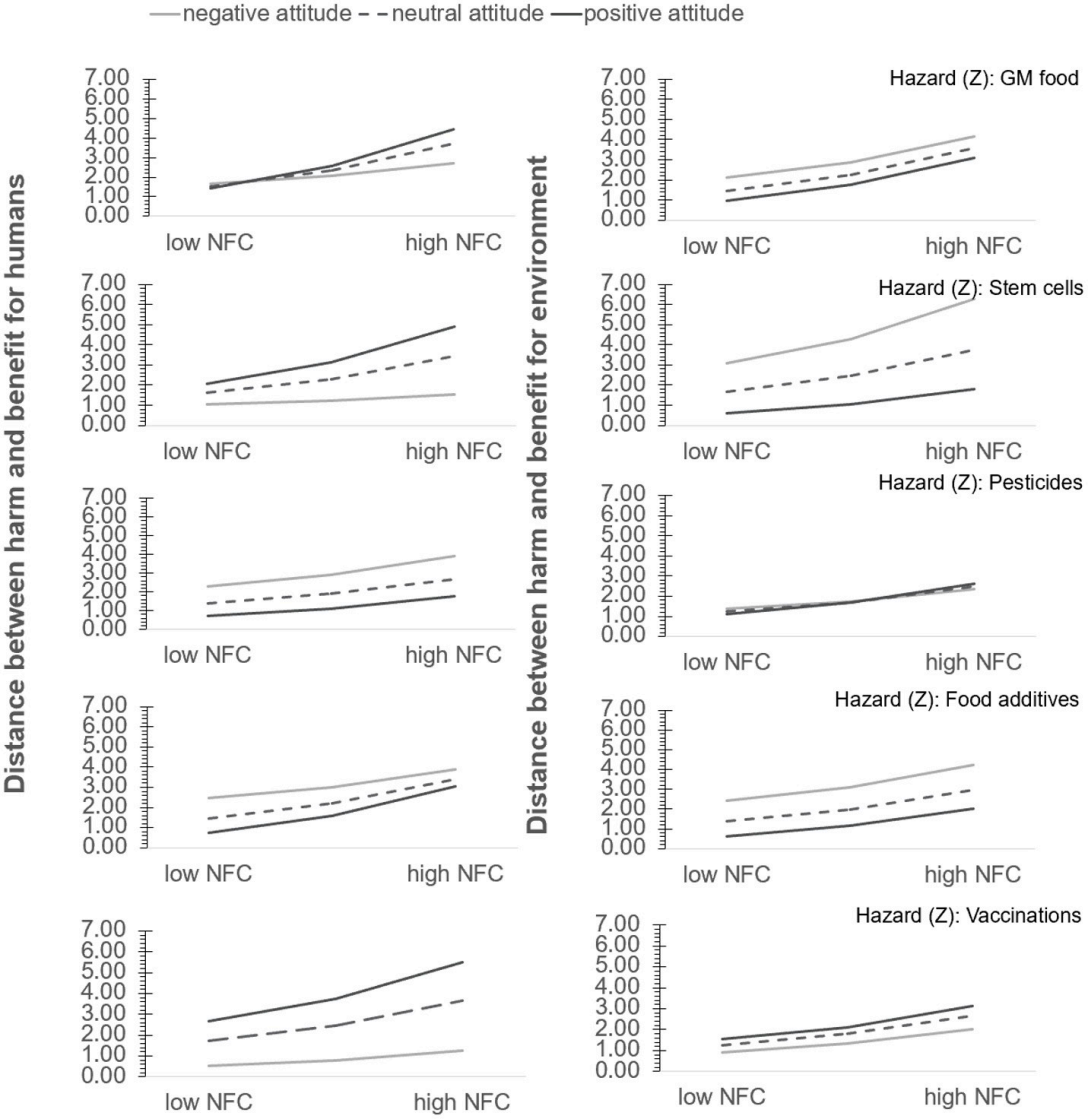
Moderated moderations of NFC (X) and the harm-benefit distance (Y). Left panel for humans and right panel for the environment.

We ran two types of models: predicting the distance between harms and benefits for humans, and the distance between harms and benefits for the environment. The model for the distance between harms and benefits in relation to humans demonstrated a good fit (*R*^*2*^ = 0.24, F[19, 16120] = 265.98, p < 0.001). The NFC as well as interaction of NFC and attitude were found to have a positive and significant impact on the inverse relation measure—the distance, while attitude negative significant. All the remaining interactions with technologies are presented in Table 3 (Model 1a). Furthermore, the test of the highest unconditional three-way interaction of NFC, attitude, and technologies on the distance accounted for 0.5% *R*^*2*^ change (F[4, 16120] = 26.08, p<0.0001). These results supported the conclusion that NFC and attitude interacted positively to predict distance between perceived harms and benefits (B = 0.01, SE = 0.001, t = 8.68, p <0.001). Most importantly the conditional effect of NFC on the harm-benefit distance, presented in Table 4, show the moderated moderation of type of technology on attitude impacted the effect of NFC on the harm-benefit distance for the human risk dimension. The conditional NFC and attitude interaction on the distance of technologies was significant and positive for GT for plant breeding (p<0.0001), stem cells (p<0.0001), food additives–“E”-numbers (p<0.0005) and vaccination (p = 0.001), but negative for pesticides (p = 0.018). Likewise, the conditional effect of NFC (as a focal predictor) on distance (an inverse-relation measure) was found to be significant and positive, regardless of attitude and technology (see Table 4). Fig 5 (left panel) presents a visualization of the three-way interaction model (conditional effect of NFC on the harm-benefit distance).

**Table 3:** Moderated moderation effects of attitude (W) and technology (Z) on the relationship between NFC and distance between perceived harms and benefits for humans (Model 1b includes demographics as controlled variables).

	Model 1a			Model 1b		
	coeff	se	Cl (95%)	coeff	se	Cl (95%)
NFC	0.01	0.01	(0.00; 0.02)*	0.01	0.01	(0.00, 0.02)*
Attitude	-0.64	0.09	(-0.82; -0.48)***	-0.64	0.10	(-0.82, -0.45)***
NFC x attitude	0.01	0.00	(0.01; 0.01)***	0.01	0.00	(0.01,0.01)***
Stem Cells	0.58	0.87	(-1.12; 2.28)	0.69	0.95	(-1.17, 2.55)
Pesticides	-1.76	0.76	(-3.26; -0.27)*	-1.58	0.83	(-3.20, 0.04)
Food Additives	-0.27	0.77	(-1.77; 1.23)	-0.01	0.83	(-1.64, 1.63)
Vaccination	-0.96	0.91	(-2.75; 0.83)	-0.62	0.99	(-2.56, 1.32)
NFC x Stem Cells	0.02	0.01	(-0.03; -0.00)*	-0.02	0.01	(-0.04, 0.00)*
NCF x Pesticides	0.03	0.01	(0.02; 0.05)***	0.03	0.01	(0.01, 0.04)***
NFC x Food Additives	0.02	0.01	(0.00; 0.03)*	0.01	0.01	(0.00, 0.03)
NFC x Vaccination	-0.01	0.01	(-0.03; 0.01)	-0.01	0.01	(-0.03, 0.01)
Attitude x Stem Cells	0.05	0.13	(-0.20; 0.29)	0.03	0.14	(-0.24, 0.30)
Attitude x Pesticides	0.60	0.13	(0.35; 0.85)***	0.57	0.14	(0.31, 0.84)***
Attitude x Food Additives	0.13	0.13	(-0.12; 0.38)	0.09	0.14	(-0.18, 0.36)
Attitude x Vaccination	0.29	0.13	(0.04; 0.54)*	0.24	0.14	(-0.03, 0.51)
NFC x Attitude x Stem Cells	0.00	0.00	(-0.00, 0.00)	0.00	0.00	(0.00, 0.00)
NFC x Attitude x Pesticides	-0.01	0.00	(-0.01; -0.01)***	-0.01	0.00	(-0.01, -0.01)***
NFC x Attitude x Food Additives	0.00	0.00	(-0.01; -0.00)***	0.00	0.00	(-0.01, 0.00)**
NFC x Attitude x Vaccination	0.00	0.0	(-0.00; 0.00)	0.00	0.00	(0.00, 0.00)
Control variables						
College education				-0.05	0.04	(-0.13, 0.04)
income				-0.25	0.04	(-0.33, -0.16)***
Gender				0.06	0.04	(-0.02, 0.15)
Age				0.01	0.00	0.01, 0.02
	*R*^*2*^ = 0.24;F(19, 16120) = 265.98***			*R*^*2*^0.25;F(23, 13856) = 205.28***		

* = p < 0.05,

** = p < 0.01,

*** = p < 0.001

Note. technology (Z) with gene technology for plant breeding as reference group.

**Table 4:** Moderated moderation for the distance between harms and benefits for humans. Conditional effect of the focal predictor at each value of the moderators.

Conditional effect of NFC (the focal predictor) at values of moderators						
Technology	Attitude	Coefficient	S.E.	t value	p value	95% CI
**GT for plant breeding**	Negative	0.0264	0.0038	7.0131	0.0000	0.0190, 0.0338
	Neutral	0.0542	0.0024	22.3140	0.0000	0.0494, 0.0589
	Positive	0.0750	0.0036	20.7270	0.0000	0.0679, 0.0821
**GT for stem cells**	Negative	0.0118	0.0047	2.5022	0.0124	0.0026, 0.0211
	Neutral	0.0455	0.0026	17.7845	0.0000	0.0405, 0.0506
	Positive	0.0708	0.0030	23.4500	0.0000	0.0649, 0.0767
**Pesticides**	Negative	0.0403	0.0033	12.0946	0.0000	0.0337, 0.0468
	Neutral	0.0322	0.0027	11.7116	0.0000	0.0268, 0.0375
	Positive	0.0261	0.0045	5.7874	0.0000	0.0172, 0.0349
**Food additives—“E”-numbers**	Negative	0.0356	0.0034	10.4933	0.0000	0.0289, 0.0422
	Neutral	0.0479	0.0027	18.0589	0.0000	0.0427, 0.0531
	Positive	0.0572	0.0043	13.1670	0.0000	0.0487, 0.0657
**Vaccination**	Negative	0.0179	0.0053	3.3476	0.0008	0.0074, 0.0284
	Neutral	0.0480	0.0029	16.7400	0.0000	0.0424, 0.0536
	Positive	0.0705	0.0027	25.6674	0.0008	0.0652, 0.0759

Note: W values (attitude) in conditional tables are the 16^th^, 50^th^, and 84^th^ percentiles.

Next, the model for distance between harms and benefits for the environment demonstrated a good fit (*R*^*2*^ = 0.21, F[19, 16120] = 219.97, p < 0.001) (see Table 5, Model 1a). The test of the highest unconditional three-way interaction of NFC, attitude, and technologies on the distance accounted for 0.3% *R*^*2*^ change (F[4, 16120] = 17.41, p<0.0001). The interaction between NFC and attitude was, however, not significant. The test of conditional NFC and attitude interaction on the distance of technologies was positive and significant for GT for stem cells (p = 0.03) and vaccination (p = 0.05), and negative and significant for pesticides (p<0.0001), but not significant for GT for plant breeding (p = 0.69) or food additives–“E”-numbers (p = 0.12). Table 6 (Model 2a) shows that the conditional effect of NFC on the distance was significant and positive, regardless of attitude and technology. See Fig 5 (right panel) for a visualization of the three-way interaction model.

**Table 5:** Moderated moderation effects of attitude (W) and technology (Z) on the relationship between NFC and distance between perceived harms and benefits for the environment (Model 2b includes demographics as controlled variables).

	Model 2a			Model 2b		
	coeff	se	Cl (95%)	coeff	se	Cl (95%)
NFC	0.05	0.01	(0.04; 0.06)***	0.05	0.01	(0.04, 0.07)**
Attitude	-0.19	0.09	(-0.37; -0.02)*	-0.11	0.10	(-0.30, 0.08)
NFC x attitude	0.00	0.00	(-0.00; 0.00)	0.00	0.00	(0.00, 0.00)
Stem Cells	1.63	0.89	(-0.12; 3.38)	2.09	0.97	(0.19, 3.99)*
Pesticides	-2.35	0.78	(-3.88; -0.81)**	-2.21	0.84	(-3.86, -0.55)**
Food Additives	0.83	0.79	(-0.72; 2.37)	1.45	0.85	(-0.21, 3.12)
Vaccination	0.61	0.94	(-1.23; 2.45)	1.42	1.01	(-0.57, 3.40)
NFC x Stem Cells	-0.03	0.01	(-0.05; -0.01)***	-0.03	0.01	(-0.05, -0.02)***
NCF x Pesticides	0.04	0.01	(0.03; 0.06)***	0.04	0.01	(0.03, 0.06)***
NFC x Food Additives	0.00	0.01	(-0.02; 0.01)	-0.01	0.01	(-0.02, 0.01)
NFC x Vaccination	-0.03	0.01	(-0.04; -0.01)**	-0.03	0.01	(-0.05, -0.01)***
Attitude x Stem Cells	0.00	0.13	(-0.26; 0.25)	-0.07	0.14	(-0.34, 0.21)
Attitude x Pesticides	0.45	0.13	(0.20; 0.71)***	0.43	0.14	(0.16, 0.70)***
Attitude x Food Additives	0.05	0.13	(-0.21; 0.31)	-0.03	0.14	(-0.30, 0.24)
Attitude x Vaccination	0.14	0.13	(-0.12; 0.39)	0.02	0.14	(-0.25, 0.29)
NFC x Attitude x Stem Cells	0.00	0.00	(-0.00; 0.00)	0.00	0.00	(0.00, 0.00)
NFC x Attitude x Pesticides	-0.01	0.00	(-0.00; -0.01)***	-0.01	0.00	(-0.01, 0.00)***
NFC x Attitude x Food Additives	0.00	0.00	(-0.00; 0.00)	0.00	0.00	(0.00, 0.00)
NFC x Attitude x Vaccination	0.00	0.00	(-0.00; 0.00)	0.00	0.00	(0.00, 0.00)
Control variables						
College education				-0.04	0.04	(-0.13, 0.05)
income				-0.20	0.04	(-0.28, -0.11)***
Gender				0.09	0.04	(0.01, 0.18)*
Age				0.02	0.00	(0.02, 0.02)***
	*R*^*2*^ = 0.21;F(19, 16120) = 219.97***			*R*^*2*^ = 0.22;F(23, 13956) = 167.31***		

* = p < 0.05,

** = p < 0.01,

*** = p < 0.001

Note. technology (Z) with gene technology for plant breeding as reference group

**Table 6:** Moderated moderation for the distance between harms and benefits for the environment. Conditional effect of the focal predictor at each value of moderators.

Conditional effect of NFC (the focal predictor) at values of moderators						
Technology	Attitude	Coefficient	S.E.	t value	p value	95% CI
**GT for plant breeding**	Negative	0.0517	0.0039	13.3488	<0.001	0.0441, 0.0592
	Neutral	0.0530	0.0025	21.2394	<0.001	0.0481, 0.0578
	Positive	0.0539	0.0037	14.5153	<0.001	0.0466, 0.0612
**GT for stem cells**	Negative	0.0248	0.0049	5.0973	<0.001	0.0152, 0.0343
	Neutral	0.0321	0.0026	12.1974	<0.001	0.0269, 0.0372
	Positive	0.0375	0.0031	12.1082	<0.001	0.0315, 0.0436
**Pesticides**	Negative	0.0794	0.0034	23.2289	<0.001	0.0727, 0.0861
	Neutral	0.0514	0.0028	18.2282	<0.001	0.0459, 0.0569
	Positive	0.0304	0.0046	6.5668	<0.001	0.0213, 0.0395
**Food additives—“E”-numbers**	Negative	0.0449	0.0035	12.8865	<0.001	0.0380, 0.0517
	Neutral	0.0395	0.0027	14.4877	<0.001	0.0341, 0.0448
	Positive	0.0354	0.0045	7.9466	<0.001	0.0267, 0.0442
**Vaccination**	Negative	0.0281	0.0055	5.1210	<0.001	0.0174, 0.0389
	Neutral	0.0347	0.0029	11.7961	<0.001	0.0290, 0.0405
	Positive	0.0397	0.0028	14.0564	0.0008	0.0341, 0.0452

Note: W values (attitude) in conditional tables are the 16^th^, 50^th^, and 84^th^ percentiles.

Consistently across all five technologies and regardless of whether the attitude was negative, neutral or positive as the NFC increases, the distance between perceived harms and benefits of technologies also increases, please see Fig 5.

The left panel presents the distances for harm-benefit judgements in relation to humans for each of the technologies. The results reveal that the greatest distance was found for GM for plant breeding, stem cells and vaccinations among those with high NFC and a favorable attitude, and for pesticides and food additives–“E”-numbers among those with high NFC and an unfavorable attitude. Interestingly, when NFC increases, harms and benefits are judged to be inversely related, even among those with a neutral attitude.

The right panel shows the distance between harms and benefits of technologies in relation to the environment. For higher levels of NFC, the greatest distance was found among the unfavorable attitude group for GM for stem cells, plant breeding, and food additives–“E”-numbers. Again, in terms of judgments related to the environment, for higher levels of NFC, the harms and benefits of technologies were also judged to be inversely related among those holding a neutral attitude.

We have repeated the above analyzes controlling for demographics variables (Model 1b and Model 2b). Moreover, the moderated moderation analyses were repeated with the dependent variable as a root squared and in log format. The main results hold whether controlling or not for demographic variables (for details please see SM Models 3–6 in Tables), as the NFC increases the distance between harm and benefits for both humans and environment increases).

## 4. Discussion

In the classical view of rational choice, a judgement of a technology or an activity’s risk and benefits would be either independent or correspond to an expectation of a positive relationship. Prior research has demonstrated that judgements of technologies’ risks and benefits are inversely related and that these judgements are based on affect. Previously researchers have investigated factors, such as expertise, as a possible exemption from affect-based judgments, however, findings are not conclusive. This paper examined the role of need for cognition (NFC) in judgements of technologies’ risk and benefits, as NFC was previously found a good predictor of rational decision making and accurate judgements, we have expected high NFC to be less prompt to inversely related judgements of technologies’ risk and benefits. Our findings, however, provide a strong support for an inverse relation (negative correlation) between judgements of risks and benefits across five technologies among individuals with a high need for cognition (NFC), which indicates a rational information processing system. Individuals with low NFC, in contrast, perceived harms and benefits for all of the technologies to be positively related.

A model of moderated moderation was utilized to evaluate the robustness of our findings, with attitude as the primary moderator and technology type as the secondary moderator. Specifically, using a three-way interaction analysis, we found that: (1) people are most prone to inverse-relation judgements when they are high on NFC; (2) inverse relation in risks and benefits evaluation is most likely among those with high NFC at all levels of attitude (from unfavorable through neutral to favorable); and (3) the attitude relation was found to be significant in all scenarios, i.e., the attitude was favorable, neutral, and unfavorable. Attitude strength and magnitude were determined to be specific to technologies and their corresponding consequences (humans and the environment). Specifically, for GT for plant breeding, stem cells, food additives–“E”-numbers and vaccination, the highest distance (inverse relation) between risks and benefits in relation to humans was found to be most likely when NFC was high and attitude was positive. For pesticides, however, the inverse relation concerning humans was most likely when NFC was high and attitude was negative. For the evaluation of environmental risks and benefits, GT for plant breeding and food additives–“E”-numbers was shown to have the highest distance when NFC was high, and no significant differences were observed among attitudinal groups. Stem cell and vaccination harm-benefit relations were the most inverse when NFC was high and attitude was positive; whereas, with pesticides, this was the case when NFC was high and attitude was negative. These results hold even when controlling for demographics and/or when dependent variable is in a squared root or log format.

Moreover, our findings might suggest a diminished role of affective evaluation in risk-benefit judgements on technologies, which is in accordance with the extant literature. In particular, it was previously reported that, regardless of favorable or unfavorable affect, technologies’ risks and benefits were found to be judged as positively related [66–68]. In a similar way, we neither find a negative correlation between risks and benefits among those with low NFC, nor do we find high distance (inverse relation) in our three-way interaction model. Regardless of attitude towards technology, the low NFC group estimated risks and benefits as positively related. It has to be mentioned that in the presented study we only measure general attitudes towards technologies (as mentioned in Method general attitudes was used in previous studies as a proxy for affect). Thus, conclusions have to be made with cautions and to understand the direct relation between affect and NFC and their impact on (inverse)relation between risk and benefit future research is needed (for example see [69]).

Our findings revealed an interesting impact of attitude magnitude and strength when NFC was high. The high NFC group, irrespective of attitude (positive, negative, or neutral attitude), judged technologies’ risks and benefits to be inversely related, which differ from findings reported by Alhakami and Slovic [2]. Alhakami and Slovic [2] identified a U-shaped relationship between affective evaluation of hazards (distance between risk and benefits), and that the distance was high for those who were positive or negative towards hazards. As the evaluation of the hazards moved into the middle of the scale towards neutral, Alhakami and Slovic reported a lower distance between risk and benefit perception. In this study, however, within the high NFC group, we found a high distance between risks and benefits when the attitude was neutral.

Our results are consistent with previous findings demonstrating that high performance in numeracy tests was associated with polarization over risks. Kahan and collaborators [70] found participants with a higher score in numeracy test to be more prompt to polarize over climate change and nuclear power risks. This result was confirmed in another study by Kahan [71], in which polarization was shown to be the greatest among those with a high score on the Cognitive Reflection Test (CRT) [29]–a task solving measurement for information processing. Kahan’s findings were in accordance with previous studies that reported polarization to be linked to a more knowledgeable public [72, 73].

It should be mentioned that, in this study, a different type of measurement of rational information processing systems—self-reporting NFC, was used. The CRT and the numeracy scale are task-solving tests have been applied in studies by Kahan and found to predict polarization over risk associated with climate change and nuclear power [70, 71]. Indeed, a debate remains regarding the adequacy of the measurements of information processing, in general, which results in criticism of the CRT and self-reported measures, such as the Need for Cognition used in this particular study. However, in numerus investigations, CRT and NFC have been found to be correlated [35, 38, 74], and both measurements were determined to lead to similar results in terms of risk polarization.

The ongoing debate about a possible explanation of polarization over risk leads to the concept of motivated reasoning. As concluded by Kahan [71], reason-based, effortful information processing enlarged the impact of motivated reasoning and “*motivated cognition (…) penetrate the form of information processing associated with Kahneman’s System 2 (…) reasoning*”. Motivated cognition, also termed motivated reasoning, refers to a tendency to adjust assessments of information for a specific reason, such as personal goals of protecting once identity (for additional details, see Kunda [75]; Taber and Lodge [76]). This explanation has been investigated primarily in relation to politically controversial issues. Specifically, in a recent study by Kahan, Peters, Dawson and Slovic [77], it was found that a politically-related task (a ban on guns) led to highly politically polarized and incorrect responses among those with the highest numeracy skills. In their study, participants were asked to make inferences from fictional experiments, in which one was politically related, i.e., carrying concealed weapons in public vs. effectiveness of a new skin treatment. Even though the data were the same in both cases, in the case of the skin treatment, as numeracy increased, the correct number of inferences increased as well, regardless of political orientation. In the case of the gun ban, political polarization increased as numeracy increased and accuracy of inferences decreased. Kahan and collaborators deemed their results to be in accordance with the Identity-Protective Cognition thesis, as it predicts that more numerate individuals “*use their quantitative-reasoning capacity selectively to conform their interpretation of the data to the result most consistent with their political outlooks*.” Kahan and colleagues discuss identity-protective cognition as a form of motivated reasoning, which can be viewed as a self-defending mechanism that drives individuals away from beliefs that do not fit within their group identity. In our study, although we did not control for political orientation as an additional factor to the cognitive explanation, the role of political polarization was found to be ambiguous [78]. Schuldt and Pearson [79], in their study on a U.S. sample, found climate change risks to be less politically polarized for racial and ethnic minorities than for Caucasians. In addition, even though not all of the technologies studied in the presented research are considered to be publicly controversial (e.g., pesticides, food additives), similar results were obtained for those that were considered to be debatable (stem cells, vaccinations, gene technology for plant breeding). In this sense, the presented results add to the current literature by indicating that polarization over technology might not necessarily be due to political orientation, but could rather be viewed from a broader perspective.

The present study is not without some limitations. First, the measure of attitude that was used as a proxy for affect. Explicit attitudes were expected to reflect respondents’ global preferences that follow from affect. In the current study, the measure of general attitude as a proxy for affect is in agreement with the definition given by the authors of the affect heuristic that ‘‘*affect means the specific quality of ‘‘goodness” or ‘‘badness” (i) experienced as a feeling state (with or without consciousness) and (ii) demarcating a positive or negative quality of a stimulus*” [80]. Finucane, Peters and Slovic [81] stated that stimuli differed in the degree to which they evoked positive and negative feelings, and that an affect can constitute a stable characteristic of a stimulus. In classic theories, attitude has been regarded as a three-component concept: affect, cognition, and behavior. In modern theories, however, many aspects of this concept are questioned, e.g., the relation between attitude and behavior, the idea of a single attitude (dual attitudes, e.g., Wilson, Lindsey, & Schooler, [82]), implicit vs. explicit attitudes and related measurements (e.g., Gawronski & Bodenhausen, [83]), and even the existence of attitudes as relatively stable mental representations (e.g., Schwartz, [84]). However, in most theories of attitudes, affect is included. Nevertheless, we consider this as a limitation of our study. In future research, various types of measurements of affect should be tested. Second, in this study we only measured the rational information processing system with the aid of NFC. It would be beneficial in the future research to include the measure of System 1 information processing and specifically including the measure of faith in intuition subscale of Rational-Experiential Inventory [34].

Indeed, the inverse relation in risk and benefit perception is assumed to originate in a rapid, heuristic, System 1 processing [20]. Kahneman and Frederick [21] pointed out that the affect heuristic is a basic mechanism that guides heuristic judgements and leads to attribute substitutions. The affect heuristic, as stated by Slovic et al. [85, 86], is a centerpiece of the experiential information processing style, as it is an automatic affect-driven mechanism. In line with this approach, [3], in their first study, found that the inverse relation between perceived risks and benefits increased when judgements were made under time constraints. As interpreted by Slovic [87], choices made under time pressure led to reduced “*opportunity for analytical thinking*”. One study showed that in evaluating familiar objects high NFC led to more reliance on cognition while high faith in intuition to more reliance on affect [69], however, no direct relation between these two measurements with risk and benefit judgments of hazards was checked.

The order of the presented materials constitutes another possible limitation of the current study. Concerning the risk questionnaire and attitude, we followed the order of previous studies. We deliberately placed NFC at the end of the list of measurements. NFC is a measure of individual differences in information processing which, in principle, are stable traits that are not likely to be influenced by previous measurements.

In conclusion, this study opens up new perspectives that facilitate a broader understanding of technologies’ risk judgements and the impact of reasoning. We found the need for cognition to be an accurate predictor of inverse relation in risk and benefit judgements. To the best of our knowledge, the current study is one of the first to focus on information processing style and inverse relation of risks and benefits in technologies’ judgements, and thus additional research is requisite. Nevertheless, as motivated reasoning currently serves as a possible explanation of technologies’ risk perception, such formulated thesis raises the critical issue of communication. Communication is especially challenging in light of the polarization over environmental risks and new technologies, and the social sciences have a fundamental role to play in understanding and overcoming this polarization. More research that focuses on cognitive components of risk perception is needed for a better understanding of the communication needs of modern technologies. Indeed, the way that we process information influences our perception of the surrounding world. Sloman and Rabb [88] recently argued that affect, reason, and individual/group representations continually interact with each other and direct people’s attitudes and opinions in an integrated manner. A question that should be addressed in future research is whether the impact of information processing on risk evaluation constitutes a universal explanation or whether it is related to socially debated issues and other social factors. The extant literature, however, lacks evidence of more broad components of technologies’ risk perception, which we suggest should be an area of active focus for future investigations.

## Supporting information

S1 Dataset (SAV)

S1 File (DOCX)
