# Supplementary material for: The inverse relation between risks and benefits: The impact of individual differences in information processing style
Source: PLoS One. 2021 Aug 9;16(8):e0255569. doi: 10.1371/journal.pone.0255569 (PMC8351923; doi:10.1371/journal.pone.0255569)
Supplement: S1 File — (DOCX) [file pone.0255569.s002.docx]

**S1 File.**

**16-item risk questionnaire by Savadori, Savio, Nicotra, Rumiati, Finucane and Slovic, 2004**

1. Dread: How much does this application frighten you? (1 = not at all; 11 = very much)

2. New: Is it a new risk or an old and familiar risk? (1 = absolutely old; 11 = absolutely new)

3. Voluntary extent of exposure to risk: To what extent people can decide to voluntarily expose himself/herself to the risk? (1 = exposure is involuntary; 11 = exposure is voluntary)

4. Personal exposure to the risk: How much you think you are personally exposed to the potential risk derived from this application? (1=not at all exposed; 11 = completely exposed)

5. Collective exposure to the risk: How many people in the world are exposed to these risks? (1 = very few people; 11 = many people)

6. Observability of the damage: To what extent is the potential damage produced by the application observable? (1 = absolutely not observable; 11 = definitely observable)

7. Severity of negative consequences: How severe are the potential negative consequences of this application? (1 = not at all severe; 11 = extremely severe)

8. Risk for future generations: To what extent does it pose a risk to future generations? (1 = risk is very low; 11 = risk is very high)

9. Personal knowledge of the risk: How precise is your personal knowledge of the risk associated with this practice? (1 = absolutely not precise; 11 = extremely precise)

10. Scientific knowledge of the risk: How precise is scientific knowledge of the risk associated with this application? (1=definite low knowledge; 11 = very high knowledge)

11. Benefits for humans: To what extent will humans benefit from this application? (1 = no benefits at all; 11 = many benefits)

12. Personal benefits: To what extent will you personally benefit from this application? (1 = no benefits at all; 11 = many benefits)

13. Benefits for the environment: To what extent will the environment benefit from this application? (1 = no benefits at all; 11 = many benefits)

14. Harm to humans: How much harm will derive from this application to humans? (1=no harm at all; 11 = very much harm)

15. Harm to environment: How much harm will derive from this application to the environment? (1 = no harm at all; 11 = very much harm)

16. Risk acceptability: To what extent do you think the risks associated with this application are acceptable to obtain the benefits? (1=not acceptable at all; 11 = definitely acceptable)

**S1 Table.** Correlation matrix for NFC and demographics.

|  | 1. | 2. | 3. |
| --- | --- | --- | --- |
| 1. NFC | - |  |  |
| 1. Age | 0.24^**^ | - |  |
| 1. College education | 0.19^**^ | 0.04^*^ | - |
| 1. Income | 0.10^**^ | 0.07^**^ | 0.21^**^ |

*Note. ** p<0.01; p<0.05;*

**S2 Table.** Benefit and harm for humans and environment means and correlation – entire sample*.*

|  | Benefit/Harm to Humans | | |  | Benefit/Harm to Environment | | |
| --- | --- | --- | --- | --- | --- | --- | --- |
|  | M benefit | M harm | Person r |  | M benefits | M harm | Person r |
| GT for plant breeding | 6.58 (2.43) | 6.08 (2.41) | -0.126** |  | 5.54 (2.51) | 6.29 (2.43) | -0.167** |
| GT for stem cells | 7.13 (2.41) | 5.45 (2.33) | -0.108** |  | 5.51 (2.42) | 5.27 (2.40) | 0.224** |
| Pesticides | 5.98 (2.38) | 7.08 (2.33) | -0.031* |  | 4.58 (2.61) | 7.35 (2.43) | -0.203** |
| Food additives | 5.51 (2.53) | 6.48 (2.42) | -0.096** |  | 4.64 (2.59) | 6.22 (2.41) | 0.048** |
| Vaccination | 7.71 (2.53) | 5.22 (2.53) | -0.260** |  | 5.71 (2.54) | 5.07 (2.49) | 0.162** |
| ** correlation is significant at 0.01 level (2-tailed); *correlation is significant at 0.05 level (2-tailed); scale from 1 to 11; N=3228; numbers | | | | | | | |

**S3 Table.** Benefit and harm for humans and environment means and correlation – entire sample*.*

| Technologies | Attitude: entire sample (N=3228) | Attitude: low NFC (N=500) | Attitude: high NFC (N=635) |
| --- | --- | --- | --- |
| GT for plant breeding | 42.86 (27.59) | 51.30 (28.09) | 44.96 (32.14) |
| GT for stem cells | 54.70 (27.86) | 55.40 (27.36) | 65.50 (29.79) |
| pesticides | 34.99 (26.22) | 49.82 (29.39) | 29.12 (26.94) |
| food additives | 35.31 (26.27) | 48.26 (28.64) | 32.49 (28.16) |
| vaccination | 62.67 (29.07) | 59.26 (27.28) | 75.67 (29.04) |
| Note: attitudes were estimated on the scale from 0 (very negative) to 100 (very positive); the number in brackets after the mean values represents SD. | | | |

**S4 Table.** t-Test Results for NFC, Attitudes towards technologies, distance between perceived technologies’ risk and benefits for humans and distance between perceived technologies’ risk and benefits of environment, for Male vs. Female respondents.

| Gender |  | N | Mean | SD | t | df | p |
| --- | --- | --- | --- | --- | --- | --- | --- |
| **NFC** | Male | 1606 | 105.92 | 18.88 | -0.03 | 3213.04 | 0.98 |
|  | Female | 1622 | 105.94 | 17.90 |  |  |  |
| **Attitude** |  |  |  |  |  |  |  |
| GT for plant breeding | Male | 1606 | 5.63 | 2.79 | 7.19 | 3226.00 | 0.00 |
|  | Female | 1622 | 4.94 | 2.69 |  |  |  |
| GT for stem cells | Male | 1606 | 6.57 | 2.82 | 2.01 | 3226.00 | 0.04 |
|  | Female | 1622 | 6.37 | 2.75 |  |  |  |
| Pesticides | Male | 1606 | 4.74 | 2.66 | 5.14 | 3226.00 | 0.00 |
|  | Female | 1622 | 4.26 | 2.56 | 7.53 |  |  |
| Food additives – “E”-numbers | Male | 1606 | 4.88 | 2.64 |  | 3226.00 | 0.00 |
|  | Female | 1622 | 4.19 | 2.57 |  |  |  |
| Vaccination | Male | 1606 | 7.20 | 2.95 | -1.22 | 3226.00 | 0.22 |
|  | Female | 1622 | 7.33 | 2.86 |  |  |  |
| **Distance humans** |  |  |  |  |  |  |  |
| GT for plant breeding | Male | 1606 | 2.54 | 2.82 | 2.28 | 3213.52 | 0.02 |
|  | Female | 1622 | 2.32 | 2.68 |  |  |  |
| GT for stem cells | Male | 1606 | 2.79 | 3.00 | 2.48 | 3192.01 | 0.01 |
|  | Female | 1622 | 2.54 | 2.73 |  |  |  |
| Pesticides | Male | 1606 | 2.20 | 2.49 | -4.31 | 3196.60 | 0.00 |
|  | Female | 1622 | 2.60 | 2.77 |  |  |  |
| Food additives – “E”-numbers | Male | 1606 | 2.38 | 2.66 | -4.22 | 3213.45 | 0.00 |
|  | Female | 1622 | 2.79 | 2.86 |  |  |  |
| Vaccination | Male | 1606 | 3.23 | 3.29 | -0.93 | 3226.00 | 0.35 |
|  | Female | 1622 | 3.34 | 3.22 |  |  |  |
| **Distance (environment)** |  |  |  |  |  |  |  |
| GT for plant breeding | Male | 1606 | 2.46 | 2.89 | -0.87 | 3226.00 | 0.39 |
|  | Female | 1622 | 2.55 | 2.96 |  |  |  |
| GT for stem cells | Male | 1606 | 1.97 | 2.52 | 3.63 | 3182.63 | 0.00 |
|  | Female | 1622 | 1.66 | 2.26 |  |  |  |
| Pesticides | Male | 1606 | 3.06 | 3.21 | -5.59 | 3204.69 | 0.00 |
|  | Female | 1622 | 3.73 | 3.52 |  |  |  |
| Food additives – “E”-numbers | Male | 1606 | 2.26 | 2.65 | -5.30 | 3187.47 | 0.00 |
|  | Female | 1622 | 2.79 | 2.98 |  |  |  |
| Vaccination | Male | 1606 | 2.06 | 2.58 | -0.09 | 3226.00 | 0.93 |
|  | Female | 1622 | 2.07 | 2.63 |  |  |  |

**S5 Table.** t-Test Results for NFC, Attitudes towards technologies, distance between perceived technologies’ risk and benefits for humans and distance between perceived technologies’ risk and benefits of environment, for responders with lower vs. higher (college or other post high school degree).

| Education |  | N | Mean | SD | t | df | p |
| --- | --- | --- | --- | --- | --- | --- | --- |
| **NFC** | lower education | 1553 | 102.40 | 17.35 | -10.79 | 3205.37 | 0.00 |
|  | higher education | 1655 | 109.28 | 18.75 |  |  |  |
| **Attitude** |  |  |  |  |  |  |  |
| GT for plant breeding | lower education | 1553 | 5.11 | 2.69 | -3.58 | 3204.76 | 0.00 |
|  | higher education | 1655 | 5.46 | 2.81 |  |  |  |
| GT for stem cells | lower education | 1553 | 6.19 | 2.79 | -5.90 | 3206.00 | 0.00 |
|  | higher education | 1655 | 6.76 | 2.75 |  |  |  |
| Pesticides | lower education | 1553 | 4.44 | 2.60 | -1.41 | 3206.00 | 0.16 |
|  | higher education | 1655 | 4.57 | 2.65 |  |  |  |
| Food additives – “E”-numbers | lower education | 1553 | 4.32 | 2.53 | -4.57 | 3205.95 | 0.00 |
|  | higher education | 1655 | 4.74 | 2.70 |  |  |  |
| Vaccination | lower education | 1553 | 6.88 | 2.94 | -7.58 | 3206.00 | 0.00 |
|  | higher education | 1655 | 7.65 | 2.82 |  |  |  |
| **Distance humans** |  |  |  |  |  |  |  |
| GT for plant breeding | lower education | 1553 | 2.28 | 2.71 | -3.03 | 3206.00 | 0.00 |
|  | higher education | 1655 | 2.58 | 2.79 |  |  |  |
| GT for stem cells | lower education | 1553 | 2.43 | 2.78 | -4.63 | 3205.87 | 0.00 |
|  | higher education | 1655 | 2.90 | 2.94 |  |  |  |
| Pesticides | lower education | 1553 | 2.33 | 2.66 | -1.52 | 3206.00 | 0.13 |
|  | higher education | 1655 | 2.47 | 2.62 |  |  |  |
| Food additives – “E”-numbers | lower education | 1553 | 2.48 | 2.78 | -2.10 | 3206.00 | 0.04 |
|  | higher education | 1655 | 2.69 | 2.75 |  |  |  |
| Vaccination | lower education | 1553 | 2.89 | 3.09 | -6.91 | 3204.13 | 0.00 |
|  | higher education | 1655 | 3.68 | 3.37 |  |  |  |
| **Distance (environment)** |  |  |  |  |  |  |  |
| GT for plant breeding | lower education | 1553 | 2.33 | 2.87 | -3.37 | 3204.02 | 0.00 |
|  | higher education | 1655 | 2.68 | 2.98 |  |  |  |
| GT for stem cells | lower education | 1553 | 1.74 | 2.40 | -1.86 | 3206.00 | 0.06 |
|  | higher education | 1655 | 1.89 | 2.39 |  |  |  |
| Pesticides | lower education | 1553 | 3.21 | 3.37 | -3.18 | 3206.00 | 0.00 |
|  | higher education | 1655 | 3.59 | 3.40 |  |  |  |
| Food additives – “E”-numbers | lower education | 1553 | 2.45 | 2.87 | -1.57 | 3206.00 | 0.12 |
|  | higher education | 1655 | 2.60 | 2.80 |  |  |  |
| Vaccination | lower education | 1553 | 1.92 | 2.51 | -3.13 | 3206.00 | 0.00 |
|  | higher education | 1655 | 2.21 | 2.68 |  |  |  |

**S6 Table.** t-Test Results for NFC, Attitudes towards technologies, distance between perceived technologies’ risk and benefits for humans and distance between perceived technologies’ risk and benefits of environment, for respondents with lower annual income (<40000 SEK) vs. higher annual income (≥40000 SEK) .

| Income |  | N | Mean | SD | t | df | p |
| --- | --- | --- | --- | --- | --- | --- | --- |
| **NFC** | lower income | 1549 | 104.55 | 18.36 | -5.17 | 2800.00 | 0.00 |
|  | higher income | 1253 | 108.17 | 18.46 |  |  |  |
| **Attitude** |  |  |  |  |  |  |  |
| GT for plant breeding | lower income | 1549 | 5.28 | 2.79 | -2.09 | 2800.00 | 0.04 |
|  | higher income | 1253 | 5.50 | 2.73 |  |  |  |
| GT for stem cells | lower income | 1549 | 6.44 | 2.86 | -2.18 | 2737.99 | 0.03 |
|  | higher income | 1253 | 6.67 | 2.69 |  |  |  |
| Pesticides | lower income | 1549 | 4.50 | 2.68 | -1.15 | 2800.00 | 0.25 |
|  | higher income | 1253 | 4.62 | 2.62 |  |  |  |
| Food additives – “E”-numbers | lower income | 1549 | 4.52 | 2.65 | -1.60 | 2800.00 | 0.11 |
|  | higher income | 1253 | 4.68 | 2.67 |  |  |  |
| Vaccination | lower income | 1549 | 7.16 | 2.96 | -3.77 | 2721.00 | 0.00 |
|  | higher income | 1253 | 7.57 | 2.84 |  |  |  |
| **Distance humans** |  |  |  |  |  |  |  |
| GT for plant breeding | lower income | 1549 | 2.48 | 2.83 | 0.23 | 2735.66 | 0.82 |
|  | higher income | 1253 | 2.45 | 2.67 |  |  |  |
| GT for stem cells | lower income | 1549 | 2.70 | 2.91 | -0.73 | 2800.00 | 0.46 |
|  | higher income | 1253 | 2.78 | 2.87 |  |  |  |
| Pesticides | lower income | 1549 | 2.49 | 2.72 | 2.46 | 2775.70 | 0.01 |
|  | higher income | 1253 | 2.25 | 2.42 |  |  |  |
| Food additives – “E”-numbers | lower income | 1549 | 2.65 | 2.86 | 0.89 | 2740.34 | 0.37 |
|  | higher income | 1253 | 2.56 | 2.68 |  |  |  |
| Vaccination | lower income | 1549 | 3.26 | 3.28 | -1.78 | 2800.00 | 0.07 |
|  | higher income | 1253 | 3.49 | 3.29 |  |  |  |
| **Distance (environment)** |  |  |  |  |  |  |  |
| GT for plant breeding | lower income | 1549 | 2.52 | 3.01 | 0.26 | 2749.05 | 0.80 |
|  | higher income | 1253 | 2.49 | 2.79 |  |  |  |
| GT for stem cells | lower income | 1549 | 1.89 | 2.48 | 0.93 | 2743.70 | 0.35 |
|  | higher income | 1253 | 1.80 | 2.31 |  |  |  |
| Pesticides | lower income | 1549 | 3.38 | 3.49 | 0.07 | 2757.80 | 0.95 |
|  | higher income | 1253 | 3.37 | 3.19 |  |  |  |
| Food additives – “E”-numbers | lower income | 1549 | 2.53 | 2.93 | 0.03 | 2754.56 | 0.98 |
|  | higher income | 1253 | 2.52 | 2.69 |  |  |  |
| Vaccination | lower income | 1549 | 2.07 | 2.63 | -0.10 | 2800.00 | 0.92 |
|  | higher income | 1253 | 2.08 | 2.56 |  |  |  |

**S7 Table.** Moderated moderation regression analysis with the squared root of absolute distance between harms and benefits for the humans (the measure of inverse relation) as the dependent variable (Model 3b includes demographics as control variables).

|  | Model 3a | | | Model 3b | | |
| --- | --- | --- | --- | --- | --- | --- |
|  | coeff | se | Cl (95%) | coeff | se | Cl (95%) |
| NFC | 0.01 | 0.00 | (0.00; 0.01)*** | 0.01 | 0.00 | (0.00; 0.01)*** |
| Attitude | -0.19 | 0.03 | (-0.25; -0.12)*** | -0.18 | 0.03 | (-0.25; -0.12)*** |
| NFC x attitude | 0.00 | 0.00 | (0.00; 0.00)*** | 0.00 | 0.00 | (0.00; 0.00)*** |
| Stem Cells | 0.16 | 0.31 | (-0.44; 0.76) | 0.19 | 0.34 | (-0.47; 0.85) |
| Pesticides | -0.37 | 0.27 | (-0.90;0.16) | -0.32 | 0.29 | (-0.89; 0.25) |
| Food Additives | 0.20 | 0.27 | (-0.34; 0.73) | 0.27 | 0.30 | (-0.31; 0.85) |
| Vaccination | -0.62 | 0.32 | (-1.26; 0.01) | -0.53 | 0.35 | (-1.22; 0.16) |
| NFC x Stem Cells | -0.01 | 0.00 | (-0.01; 0.00) | -0.01 | 0.00 | (-0.01; 0.00) |
| NCF x Pesticides | 0.01 | 0.00 | (0.00; 0.01)** | 0.01 | 0.00 | (0.00; 0.01)** |
| NFC x Food Additives | 0.00 | 0.00 | (0.00; 0.01) | 0.00 | 0.00 | (0.00; 0.01) |
| NFC x Vaccination | 0.00 | 0.00 | (-0.01; 0.01) | 0.00 | 0.00 | (-0.01; 0.01) |
| Attitude x Stem Cells | 0.02 | 0.05 | (-0.07; 0.11) | 0.01 | 0.05 | (-0.08; 0.11) |
| Attitude x Pesticides | 0.14 | 0.05 | (0.05; 0.23)** | 0.13 | 0.05 | (0.04; 0.23)** |
| Attitude x Food Additives | -0.01 | 0.05 | (-0.10; 0.07) | -0.02 | 0.05 | (-0.12; 0.07) |
| Attitude x Vaccination | 0.14 | 0.04 | (0.05; 0.23)** | 0.13 | 0.05 | (0.03; 0.22)** |
| NFC x Attitude x Stem Cells | 0.00 | 0.00 | (0.00; 0.00) | 0.00 | 0.00 | (0.00; 0.00) |
| NFC x Attitude x Pesticides | 0.00 | 0.00 | (0.00; 0.00)*** | 0.00 | 0.00 | (0.00; 0.00)*** |
| NFC x Attitude x Food Additives | 0.00 | 0.00 | (0.00; 0.00) | 0.00 | 0.00 | (0.00; 0.00) |
| NFC x Attitude x Vaccination | 0.00 | 0.00 | (0.00; 0.00) | 0.00 | 0.00 | (0.00; 0.00) |
| Control variables | | | | | | |
| College education |  |  |  | 0.00 | 0.02 | (-0.03; 0.03) |
| Income |  |  |  | -0.06 | 0.02 | (-0.09; -0.03)*** |
| gender |  |  |  | 0.03 | 0.02 | (0.00; 0.06)* |
| Age |  |  |  | 0.00 | 0.00 | (0.00; 0.01)*** |
|  | *R^2^*=0.20; F(19, 16120)=216.60*** | | | *R^2^*=0.21; F(23, 13936)=164.65*** | | |
| *∗ = p < 0.05, ∗∗ = p < 0.01, ∗∗∗ = p < 0.001* | | | | | | |

**S8 Table.** Moderated moderation regression analysis with the squared root of absolute distance between harms and benefits for the environment (the measure of inverse relation) as the dependent variable (Model 4b includes demographics as control variables).

|  | Model 4a | | | Model 4b | | |
| --- | --- | --- | --- | --- | --- | --- |
|  | coeff | se | Cl (95%) | coeff | se | Cl (95%) |
| NFC | 0.02 | 0.00 | (0.01; 0.02)*** | 0.02 | 0.00 | (0.01; 0.02)*** |
| Attitude | -0.06 | 0.03 | (-0.12; 0.00) | -0.03 | 0.04 | (-0.10; 0.04) |
| NFC x attitude | 0.00 | 0.00 | (0.00; 0.00) | 0.00 | 0.00 | (0.00; 0.00) |
| Stem Cells | 0.60 | 0.32 | (-0.02; 1.23) | 0.80 | 0.35 | (0.12; 1.48)* |
| Pesticides | -0.41 | 0.28 | (-0.96; 0.14) | -0.35 | 0.30 | (-0.95; 0.24) |
| Food Additives | 0.43 | 0.28 | (-0.12; 0.99) | 0.65 | 0.31 | (0.05; 1.25) |
| Vaccination | -0.04 | 0.34 | (-0.70; 0.62) | 0.17 | 0.36 | (-0.54; 0.89) |
| NFC x Stem Cells | -0.01 | 0.00 | (-0.02; 0.00) | -0.01 | 0.00 | (-0.02; -0.01)** |
| NCF x Pesticides | 0.01 | 0.00 | (0.00; 0.01) | 0.01 | 0.00 | (0.00; 0.01)** |
| NFC x Food Additives | 0.00 | 0.00 | (-0.01; 0.00) | 0.00 | 0.00 | (-0.01; 0.00) |
| NFC x Vaccination | -0.01 | 0.00 | (-0.01; 0.00) | -0.01 | 0.00 | (-0.01; 0.00) |
| Attitude x Stem Cells | -0.01 | 0.05 | (-0.10; 0.08) | -0.04 | 0.05 | (-0.14; 0.06) |
| Attitude x Pesticides | 0.08 | 0.05 | (-0.01; 0.17) | 0.07 | 0.05 | (-0.03; 0.17) |
| Attitude x Food Additives | -0.02 | 0.05 | (-0.12; 0.07) | -0.05 | 0.05 | (-0.15; 0.05) |
| Attitude x Vaccination | 0.08 | 0.05 | (-0.02; 0.17) | 0.04 | 0.05 | (-0.06; 0.14) |
| NFC x Attitude x Stem Cells | 0.00 | 0.00 | (0.00; 0.00) | 0.00 | 0.00 | (0.00; 0.00) |
| NFC x Attitude x Pesticides | 0.00 | 0.00 | (0.00; 0.00)** | 0.00 | 0.00 | (0.00; 0.00) |
| NFC x Attitude x Food Additives | 0.00 | 0.00 | (0.00; 0.00) | 0.00 | 0.00 | (0.00; 0.00) |
| NFC x Attitude x Vaccination | 0.00 | 0.00 | (0.00; 0.00) | 0.00 | 0.00 | (0.00; 0.00) |
| Control variables | | | | | | |
| College education |  |  |  | 0.00 | 0.02 | (-0.03; 0.03) |
| Income |  |  |  | -0.03 | 0.02 | (-0.06; 0.00) |
| gender |  |  |  | 0.03 | 0.02 | (0.00; 0.06) |
| Age |  |  |  | 0.01 | 0.00 | (0.00; 0.01)*** |
|  | *R^2^*=0.17; F(19, 16120)=177.92*** | | | *R^2^*=0.18; F(23, 13936)=133.69*** | | |
| *∗ = p < 0.05, ∗∗ = p < 0.01, ∗∗∗ = p < 0.001* | | | | | | |

**S9 Table.** Moderated moderation regression analysis with the log of absolute distance between harms and benefits for the humans (the measure of inverse relation) as the dependent variable variable (Model 5b includes demographics as control variables).

|  | Model 5a | | | Model 5b | | |
| --- | --- | --- | --- | --- | --- | --- |
|  | coeff | se | Cl (95%) | coeff | se | Cl (95%) |
| NFC | 0.00 | 0.00 | (0.00; 0.00)*** | 0.00 | 0.00 | (0.00; 0.00)*** |
| Attitude | -0.06 | 0.01 | (-0.08; -0.04)*** | -0.06 | 0.01 | (-0.08; -0.04)*** |
| NFC x attitude | 0.00 | 0.00 | (0.00; 0.00)*** | 0.00 | 0.00 | (0.00; 0.00)*** |
| Stem Cells | 0.06 | 0.11 | (-0.15; 0.26) | 0.07 | 0.12 | (-0.15; 0.30) |
| Pesticides | -0.11 | 0.09 | (-0.29; 0.07) | -0.10 | 0.10 | (-0.29; 0.10) |
| Food Additives | 0.08 | 0.09 | (-0.10; 0.26) | 0.11 | 0.10 | (-0.09; 0.31) |
| Vaccination | -0.22 | 0.11 | (-0.44; -0.01)* | -0.19 | 0.12 | (-0.43; 0.05) |
| NFC x Stem Cells | 0.00 | 0.00 | (0.00; 0.00) | 0.00 | 0.00 | (0.00; 0.00) |
| NCF x Pesticides | 0.00 | 0.00 | (0.00; 0.00)*** | 0.00 | 0.00 | (0.00; 0.00)*** |
| NFC x Food Additives | 0.00 | 0.00 | (0.00; 0.00) | 0.00 | 0.00 | (0.00; 0.00) |
| NFC x Vaccination | 0.00 | 0.00 | (0.00; 0.00) | 0.00 | 0.00 | (0.00; 0.00) |
| Attitude x Stem Cells | 0.01 | 0.02 | (-0.02; 0.04) | 0.00 | 0.02 | (-0.03; 0.04) |
| Attitude x Pesticides | 0.04 | 0.02 | (0.01; 0.07)*** | 0.04 | 0.02 | (0.01; 0.07)*** |
| Attitude x Food Additives | -0.01 | 0.02 | (-0.04; 0.02) | -0.01 | 0.02 | (-0.04; 0.02) |
| Attitude x Vaccination | 0.05 | 0.02 | (0.02; 0.08)*** | 0.05 | 0.02 | (0.01; 0.08)*** |
| NFC x Attitude x Stem Cells | 0.00 | 0.00 | (0.00; 0.00) | 0.00 | 0.00 | (0.00; 0.00) |
| NFC x Attitude x Pesticides | 0.00 | 0.00 | (0.00; 0.00)*** | 0.00 | 0.00 | (0.00; 0.00)*** |
| NFC x Attitude x Food Additives | 0.00 | 0.00 | (0.00; 0.00) | 0.00 | 0.00 | (0.00; 0.00) |
| NFC x Attitude x Vaccination | 0.00 | 0.00 | (0.00; 0.00) | 0.00 | 0.00 | (0.00; 0.00) |
| Control variable | | | | | | |
| College education |  |  |  | 0.00 | 0.01 | (-0.01; 0.01) |
| Income |  |  |  | -0.02 | 0.01 | (-0.03-0.01)*** |
| Gender |  |  |  | 0.01 | 0.01 | (0.00; 0.02)* |
| Age |  |  |  | 0.00 | 0.00 | (0.00; 0.00)*** |
|  | *R^2^*=0.20; F(19, 16120)=217.60*** | | | *R^2^*=0.21; F(23, 13936)=164.65*** | | |
| *∗ = p < 0.05, ∗∗ = p < 0.01, ∗∗∗ = p < 0.001* | | | | | | |

**S10 Table.** Moderated moderation regression analysis with the log of absolute distance between harms and benefits for the environment (the measure of inverse relation) as the dependent variable variable (Model 6b includes demographics as control variables).

|  | Model 6a | | | Model 6b | | |
| --- | --- | --- | --- | --- | --- | --- |
|  | coeff | se | Cl (95%) | coeff | se | Cl (95%) |
| NFC | 0.01 | 0.00 | (0.00; 0.01)*** | 0.01 | 0.00 | (0.01; 0.01)*** |
| Attitude | -0.02 | 0.01 | (-0.04; 0.00) | -0.01 | 0.01 | (-0.03; 0.01) |
| NFC x attitude | 0.00 | 0.00 | (0.00; 0.00) | 0.00 | 0.00 | (0.00; 0.00) |
| Stem Cells | 0.20 | 0.11 | (-0.01; 0.42) | 0.27 | 0.12 | (0.03; 0.50)* |
| Pesticides | -0.12 | 0.10 | (-0.31; 0.06) | -0.10 | 0.10 | (-0.31; 0.10) |
| Food Additives | 0.15 | 0.10 | (-0.04; 0.34) | 0.22 | 0.10 | (0.02; 0.43)* |
| Vaccination | -0.02 | 0.11 | (-0.24; 0.21) | 0.06 | 0.12 | (-0.18; 0.30) |
| NFC x Stem Cells | 0.00 | 0.00 | (-0.01; 0.00)*** | 0.00 | 0.00 | (-0.01; 0.00)*** |
| NCF x Pesticides | 0.00 | 0.00 | (0.00; 0.00)*** | 0.00 | 0.00 | (0.00; 0.00)*** |
| NFC x Food Additives | 0.00 | 0.00 | (0.00; 0.00) | 0.00 | 0.00 | (0.00; 0.00) |
| NFC x Vaccination | 0.00 | 0.00 | (0.00; 0.00) | 0.00 | 0.00 | (-0.01; 0.00)* |
| Attitude x Stem Cells | 0.00 | 0.02 | (-0.03; 0.03) | -0.01 | 0.02 | (-0.05; 0.02) |
| Attitude x Pesticides | 0.02 | 0.02 | (-0.01; 0.06) | 0.02 | 0.02 | (-0.01; 0.06) |
| Attitude x Food Additives | -0.01 | 0.02 | (-0.04; 0.02) | -0.02 | 0.02 | (-0.05; 0.02) |
| Attitude x Vaccination | 0.03 | 0.02 | (0.00; 0.06) | 0.01 | 0.02 | (-0.02; 0.05) |
| NFC x Attitude x Stem Cells | 0.00 | 0.00 | (0.00; 0.00) | 0.00 | 0.00 | (0.00; 0.00) |
| NFC x Attitude x Pesticides | 0.00 | 0.00 | (0.00; 0.00)*** | 0.00 | 0.00 | (0.00; 0.00)*** |
| NFC x Attitude x Food Additives | 0.00 | 0.00 | (0.00; 0.00) | 0.00 | 0.00 | (0.00; 0.00) |
| NFC x Attitude x Vaccination | 0.00 | 0.00 | (0.00; 0.00) | 0.00 | 0.00 | (0.00; 0.00) |
| Control variables | | | | | | |
| College education |  |  |  | 0.00 | 0.01 | (-0.01; 0.01) |
| Income |  |  |  | -0.01 | 0.01 | (-0.02; 0.00) |
| Gender |  |  |  | 0.01 | 0.01 | (0.00; 0.02) |
| Age |  |  |  | 0.00 | 0.00 | (0.00; 0.00)*** |
|  | *R^2^*=0.18; F(19, 16120)=280.68*** | | | *R^2^*=0.18; F(23, 13936)=135.97*** | | |
| *∗ = p < 0.05, ∗∗ = p < 0.01, ∗∗∗ = p < 0.001* | | | | | | |
